# Prevalence and intensity of *Schistosoma mansoni* infections among schoolchildren attending primary schools in an urban setting in Southwest, Ethiopia

**DOI:** 10.1186/s13104-017-3023-9

**Published:** 2017-12-04

**Authors:** Mitiku Bajiro, Daniel Dana, Bruno Levecke

**Affiliations:** 1 School of Medical Laboratory Sciences, Faculty Health Sciences, Institute of Health, Jimma University, Jimma, Ethiopia; 2 Department of Virology, Parasitology and Immunology, Faculty of Veterinary Medicine, Ghent University, Merelbeke, Belgium

**Keywords:** *Schistosoma mansoni*, Prevalence, Infection intensity, Urban settings, Jimma Town

## Abstract

**Objective:**

To determined both prevalence and intensity of *Schistosoma mansoni* infections among schoolchildren attending primary schools in Jimma town, an urban setting, Southwest, Ethiopia.

**Results:**

The prevalence of *S. mansoni* infections was 8.4%. *S. mansoni* infections were found in all 17 schools, but the school prevalence ranged from 1.7 to 26.7%. This variation in prevalence could be explained by the proximity of the schools to the river crossing the town and water bodies near the schools. Boys were more infected compared to girls (*χ* ^2^ = 31.587, *P* value = 0.001; 95% CI), and the infection rate increased as a function of age (*χ* ^2^ = 21.187; *P* value = 0.001; 95 %CI). The majority of the infection intensities were of low intensity (57%), the mean number of eggs per stool equal to 17 eggs per gram of stool. Based on the prevalence (8.4%) school children in Jimma Town is considered as a low risk of morbidity caused by *S. mansoni* (prevalence ≤ 10% according to WHO threshold), for which it is recommended to implement MDA once every 3 years which should be supplemented with health information to create awareness about Schistosomiasis transmission. Male students were more infected than females with majority of the infection intensity were low.

**Electronic supplementary material:**

The online version of this article (10.1186/s13104-017-3023-9) contains supplementary material, which is available to authorized users.

## Introduction

Schistosomiasis is water-born parasitic disease next malaria and neglected tropical parasitic disease caused by blood flukes of the genus *Schistosoma* [1, 2]. It is estimated that Schistosomiasis accounts for 15,000–280,000 deaths per year and a total of 1.7–4.5 million disability adjusted life years lost, the majority of the disease burden affecting the African continent [3–5]. For example, Africa accounts for more than 90% of the infections occurring worldwide [6]. In Ethiopia the prevalence of *S. mansoni* infection was reported as high as 90% among school children [7]. Current means of controlling Schistosomiasis is mainly based on the administration of praziquantel to groups at risk schoolchildren, often without any prior diagnosis. The frequency of such large-scale de-worming programs is mainly based on the prevalence, Mass drug administration (MDA) being recommended every year when the prevalence in an untreated is at least 50%, once every 2 years when the prevalence is at least 10% but not higher than 50%, and once every 3 years when the prevalence does not exceeds 10% [8]. Although, Schistosomiasis is considered as public health problem in rural populations [9], which can be explained by increased exposure to water through different activities such as, agriculture, population movements, large water management, lack of safe water and inadequate sanitation and others [10–12]. One of the major factors for urban Schistosomiasis is due to the movements of infected rural population to urban area to search more attractive job opportunities in the urban areas [13]. Urban Schistosomiasis has been also reported in urban cities of African countries; Ibadan, Nigeria [14], Kisumu, Kenya [15] and Addis Ababa, Ethiopia [16] depending on the availability of intermediate host in water bodies contaminated with the infective stage. There is evidence that *S. mansoni* infection is in Jimma Town, an urban setting in Southern Ethiopia (from case reports in hospitals; presence of intermediate hosts in water crossing the Town), however, there is no documents or published data that reports about the prevalence and intensity of infections of *S. mansoni* in Jimma town, hence it remains unclear whether it should be included in MDA program. Therefore, the objective of this study was to assess the prevalence and the intensity of *S. mansoni* infections among schoolchildren in Jimma Town.

## Main texts

### Methods

#### Study area and study population

The study was conducted in Jimma Town, which is located approximately 350 km Southwest of the capital Addis Ababa at a latitude and longitude of 7°40′N36°50′E and at 1720–2010 m above sea level. The area is characterized by a semi-arid type of climate with an average annual rainfall of 800–2500 mm. The mean daily temperature is 19 °C, but ranges from 12 to 30 °C. Our study focused on schoolchildren of 5–19 years of years. In Jimma Town, there were 17 primary schools hosting a total of 23,492 children of all age groups of interest. The female/male ratio across the different schools was approximately 1:1 (Report Document 2011/2012 of Jimma Education Bureau). There is Awetu River which cross in the middle Jimma town and community use this river for different domestic purpose as well as school children have contact at different sites while passing through the town. There are also other rivers such as Kito, Kaba close to schools and other streams very close to schools (Additional file 1: Figure S1). These water sources are potentially source of infection for *S. mansoni*, as they harbor the snail intermediate host, which were tested in the laboratory and hatch cercariae which is infective stage (Alemu Y, unpublished data).

#### Study design

From February to March 2014, a cross-sectional study was conducted to assess prevalence and infection intensity of *S. mansoni* among primary schools in Jimma Town. To this end, a total of 17 primary schools in Jimma Town were included in the study. To include all primary school in Jimma town, we use at least 60 school children were sampled from each school, which result in total sample size of 1000 school children. At each school students were stratified into three age classes (5–9, 10–13 and 14–19 years) and selected by using simple random sampling techniques using students’ roster as sample frame from selected classes. From each age class at least 20 subjects were randomly selected to include students with different age classes, which result in at least 60 subjects per school. At the same time the age classification is also used to determine the variation of *S. mansoni* infections among the age classes. One stool sample per child was screened 24 h after collection applying a single Kato-Katz thick smear to quantify the eggs of *S. mansoni* eggs [17].

#### Data processing and Statistical analysis

Data were coded, entered and cleaned by using “Epi Info”. The processing and analysis of the data were carried out using SPSS version 20.0. The Prevalence of *S. mansion* infection was presented in percent. Prevalence of *S. mansoni* was calculated, for both sexes, three age groups, each of 17 schools and the mean fecal egg counts (FEC) for each schools also calculated which is expressed as eggs Per gram of stool (EPG) separately. Infection intensity was classified into low, moderate and high based on the thresholds described by the WHO guidelines (low: 1 EPG ≤ FEC < 99 EPG; moderate: 100 EPG ≤ FEC < 399 EPG; and high ≥ 400 EPG) [18]. The association of *S. mansoni* infection with age groups and sexes was statistically tested using, Chi square and *P value* < 0.05 was statistically significant.

### Results

#### Socio-demographic characteristics study participants

A total of 1000 schoolchildren (501 males and 499 females) from grade 1 to 8 and age group from 5 to 19 were involved from 17 primary schools in Jimma town. Majority of the participants involved in this study 375 (37.5%) were selected from the age group between 10–13% (Table 1).

**Table 1:** Socio-demographic characteristics and prevalence of *S. mansoni* among school children attending primary schools in Jimma town, an urban setting, southwest Ethiopia, 2014

Variables	*S. mansoni* infection status		Total (%)	*χ* ^2^	*P* value
	No. of positive (%)	No. of negative (%)			
Sex					
Male	67 (13.4)	434 (86.6)	501 (50.1)	31.587	0.001
Female	17 (3.4)	482 (96.6)	499 (49.9)		
Age (years)					
5–9	11 (3.5)	303 (96.5)	314 (31.4)	21.187	0.001
10–13	31 (8.3)	344 (91.7)	375 (37.5)		
14–19	42 (13.5)	269 (86.5)	311 (31.1)		

#### Prevalence and intensity of *S. mansoni* infection

**Fig. 1:**
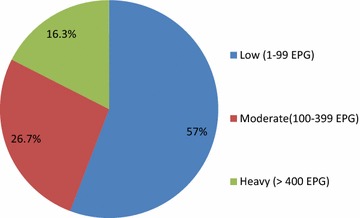
Infection intensity of *S. mansoni* infection among schoolchildren attending primary schools in Jimma town, an urban setting, southwest Ethiopia, 2014

Eggs of *S. mansoni* were detected in 84 out of the 1000 School children screened (8.4%). *S. mansoni* infections were reported in all 17 schools (Table 2). However, there was a large variation in prevalence between schools, ranging from 1.7 to 26.7%. This difference may be due to the proximity of the river crossing the town and water body close to the schools. Boys were more infected compared to girls, which is statically significant (*χ* ^2^ = 31.587, *P* value = 0.001; 95% CI) this may be due to swimming behavior of the boys in which they went away from home and playing in the fields and taking bath in infected water and the infection rate increased as a function of age 5–9, P = 3.5%, 10–13, P = 8.3%, and 14–19, P = 13.5% (*χ* ^2^ = 21.187; *P* value = 0.001; 95% CI) (Table 1) and the infection rate increased with increase age this may be because of the chance of low age group away from home and exposed to infested water with infective stage is less but the older age group they may away from home and play in the field and swimming in water bodies contaminated with infective stage of *S. mansoni* is higher in higher age group when compared with lower age group. Majorities (57%) of the infection intensity were low, among the infected children with mean fecal egg count 17 EPG (Fig. 1).

**Table 2:** The prevalence and infection intensity (mean of fecal egg counts (FEC) and proportion of low, moderate and high levels of infection intensity) across 17 schools in Jimma town, an urban setting, Southwest Ethiopia, 2014

	N (Sampled children)	Prevalence (%)	Mean FEC (EPG)	Level infection intensity		
				Low	Moderate	High
School						
1	57	1.8	1	1.8	0.0	0.0
2	60	6.7	7	5.0	1.7	0.0
3	50	4.0	16	2.0	0.0	2.0
4	60	3.3	1	3.3	0.0	0.0
5	60	3.3	1	3.3	0.0	0.0
6	56	7.1	16	3.6	1.8	1.8
7	60	10.0	19	8.3	0.0	1.7
8	60	11.7	14	6.7	5.0	0.0
9	60	3.3	2	3.3	0.0	0.0
10	57	7.0	31	1.8	1.8	3.5
11	60	5.0	14	1.7	1.7	1.7
12	60	3.3	1	3.3	0.0	0.0
13	60	1.7	14	0.0	0.0	1.7
14	60	23.3	20	16.7	6.7	0.0
15	60	11.7	6	11.7	0.0	0.0
16	60	13.3	30	5.0	6.7	1.7
17	60	26.7	98	6.7	13.3	6.7

### Discussions

Different studies have been reported epidemiology of *S. mansoni* infection in different countries of Africa including Ethiopia and recently *S. mansoni* infection was reported from unexpected areas including urban settings due to resettlement, expansion of urban area, water development projects for hydroelectric dams, irrigations, population movement and unsafe water and inadequate sanitation [10, 12, 14–16, 19].

The prevalence *S. mansoni* infection among the school children in Jimma Town; an urban setting was 8.4%. The prevalence in this study was lower than those studies reported from Ethiopia in urban settings 89.9% in Saja Town Northwest Ethiopia [7], 79.5 and 75% in Saja and Ewket Amba primary schools in Amahara region [20], 23.9% in Suburbs of Mekelle city [21], 23.9% from Gorgora Town elementary school Northwest Ethiopia [22], 67.6% Horro Guduru Wollega [23], 81.3% from Wolaita [24], 74.9% from Wondo Genet [25], 21.2% from Southeast of Lake Langano [26], 37.9% in Gonder [27], 33.7 and 15.9% Northern Gonder [28, 29], 67.95 and 73.9% from Southern Tigray [30], 89.6% from Kemissie, 59.9% Wondo Genet and 31.6% Sille-Elgo from geographically apart endemic localities of Ethiopia [31], 24% from Manna District Jimma Zone [32]. The difference might be due to the baseline endemicity of the parasite in the areas, the climate of the areas, the study design, sampling techniques and sample size.

Higher prevalence of *S. mansoni* was reported in our study area than the reports from different localities of Ethiopia including Jimma Zone, 2.1% [33], 5.95% from different water sources of Tigray region [34], 0.8% from Amibera District of Afar [35] and 1.3% from University of Gonder community school [36]. These differences may be due to the methodology, sample size used, variation in geographical location.

The prevalence in our study is lower than those reported from Brazil (14.4%) [37], Ghana (19.8%) [38], Northwest Tanazania (64.3%) [39], Agaie, Niger state, Nigeria 12.1% [40], 27.8 and 35% from Uganda [41, 42] respectively and higher than the one reported from Nigeria 5.3% [43], 4.6% from Jos Nigeria 4.6% [44] and 1.5% from Bamako, Mali [19]. The possible differences may be due to long time endemicity of study area, different geographical and ecological variations, study design, sampling techniques, sample size.

The majority (57%) of infection intensity were classified as low infection intensity which is similar with the one reported from Tumuga and Waja Southern Tigray Regional State [30], Surbs of Mekelle city [21], different water sources of Tigray [34] Southeast of Lake Langano [26], Sille-Elgo, Gofa Zone Southern Nation and Nationalities of Regional State [31], Northwestern Tanzania [39] and North Ghana [38].

The infection intensity of present study is different from the one reported from Horro Guduru Wollega Zone [23], Saja Town Northeast Ethiopia [7], Saja and Ewket Ameba primary school [20], Wondo Genet [25, 31], Kemissie Amahara administrative region Oromia Zone [31] and Jija District of Uganda [42] with moderate and heavy infection. This difference may be due to repeated exposure of school children to water bodies infested with infective stage.

In this study male participants were more infected than the female with prevalence of 13.6 and 3.6%; which is similar to those reports from different localities of Ethiopia, Horro Guduru Wollega [23], Wondo Genet [25], Suburbs of Mekelle city, Tigray [21], Sanja area, Amahara Regional state [20], Kemissie, Wondo Genet and Sille-Elgo of different geographically separated area [31], Different Water Source Users in Tigray, Northern Ethiopia [34], Amibera District, Afar [35] and Agaie, Niger state of Nigeria [40], Jos, Nigeria [44] and different from the Saja Town, Northwest Ethiopia [7] and North western Tanzania [39] in which females were more infected than males.

Increasing prevalence was reported in the three age classes with highest prevalence was observed 14–18 years which is similar with one reported Tumuga and Waja Tigray regional state [30] and different from the other reports in which age 10–14 years age group are more infected from Saja area, Amahara region, Ethiopia [20], Horro Guduru Wollega [23], Suburbs of Mekelle city, Tigray and Different Water Source Users in Tigray [21, 34], Wondo Genet [25], Sille-Elgo Gofa zone of southern parts of Ethiopia [31] and Jos, Nigeria [44] and age group 5–9 years were more infected reports from Saja primary school [20] and Wondo Genet and Kemissie from different geographically located parts of Ethiopia [31].

### Conclusion

Based on the results (8.4%) school children in Jimma Town is considered as a low risk of morbidity caused by *S. mansoni* according to WHO threshold, for which it is recommended to implement MDA once every 3 years supplemented with health information to create awareness about Schistosomiasis transmission. Furthermore we recommend longitudinal study which might give much higher in prevalence of *S. mansoni* due to expected increase of intermediate host and stagnant water pools since the present study was done during dry season.

## Limitation

We didn’t take into consideration environmental/demographic risky factors such as drinking water sources, availability of latrines, religion, socioeconomic status, geographical location of the children’s, bathing sources.

## Additional file

**Additional file 1: Figure S1.** Map of Jimma town indicating location of primary schools included in the study in relation to water body in an urban setting, southwest Ethiopia, 2014. The source of map is Google Earth after taking the ordinates of the schools by apparatus GIS (geographical information system).

## Abbreviations

EPG: egg per gram
FEC: fecal egg count
MDA: Mass drug administration
WHO: World Health Organization
