# Supplementary figures and images for: Prevalence and intensity of Schistosoma mansoni infections among schoolchildren attending primary schools in an urban setting in Southwest, Ethiopia
Source: BMC Res Notes. 2017 Dec 4;10:677. doi: 10.1186/s13104-017-3023-9 (PMC5716059; doi:10.1186/s13104-017-3023-9)

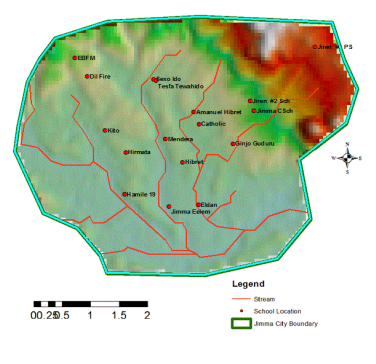

Supplement: Supplementary file 1 — Additional file 1: Figure S1. Map of Jimma town indicating location of primary schools included in the study in relation to water body in an urban setting, southwest Ethiopia, 2014. The source of map is Google Earth after taking the ordinates of the schools by apparatus GIS (geographical information system). [file 13104_2017_3023_MOESM1_ESM.docx]
